# Prospective observational study on clinical and epidemiological profile of adult patients presenting to the emergency department with suspected upper gastrointestinal bleed

**DOI:** 10.1186/s12873-023-00885-9

**Published:** 2023-09-19

**Authors:** Alok Raj, Nidhi Kaeley, Hari Prasad, Itish Patnaik, Yogesh Bahurupi, Shrirang Joshi, Krishna Shukla, Santosh Galagali, Sanket Patel

**Affiliations:** 1 Department of Emergency Medicine, All India Institute of Medical Sciences Rishikesh, Rishikesh, 249203 Uttarakhand India; 2 Department of Gastroenterology, All India Institute of Medical Sciences Rishikesh, Rishikesh, 249203 Uttarakhand India; 3 Department of Community and Family Medicine, All India Institute of Medical Sciences Rishikesh, Rishikesh, 249203 Uttarakhand India; 4 Department of Emergency Medicine, Nootan Medical College, Gujarat, 384315 India

**Keywords:** Emergency, Gastrointestinal endoscopy, Hematemesis, Melena, Portal hypertension

## Abstract

**Background and objective:**

Bleeding from the upper gastrointestinal (GI) tract is one of the common medical emergencies. In this study, we assessed patients’ socio-demographic and clinical characteristics and the association of clinical characteristics with treatment outcomes among patients with suspected upper gastrointestinal bleed (UGIB) presenting to the emergency department (ED). At present, there is a scarcity of data on UGIB in Northern part of India.

**Material and method:**

The study was a single-center, prospective observational study conducted at an urban tertiary care center. Consecutive patients with suspected UGIB were enrolled in the study from August 2020 to February 2022. A detailed history was obtained, including demographic data such as age and sex, presenting complaints, history of presenting illness, history related to co-morbidities, addiction, and drug history. Pre-endoscopic Rockall and Glasgow-Blatchford Score were calculated for each patient. The patients were subsequently followed up till discharge from the hospital. The final outcomes with regard to mortality, need for blood transfusion, length of emergency department stay, and discharge were noted.

**Result:**

141 patients were included in the study. The mean age of the patients with suspected UGIB was 48 ± 14 years. 115 (81.6%) patients were male. The most common co-morbidity was chronic liver disease (40;28.4%). The most frequent presenting complaint in this study was hematemesis (96; 68.1%), followed by melena (76;53.9%). The mean (Standard Deviation, SD) of the Rockall Score was 2.46 ± 1.75. The mean (SD) of the Glasgow Blatchford Score was 12.46 ± 3.15 in patients with UGIB.

**Conclusion:**

In our study, hematemesis was the most prevalent symptom of suspected UGIB, followed by melena. Portal hypertension was the most common cause of UGIB. Most frequent comorbidities in patients suspected of UGIB were alcohol intake, Nonsteriodal Antiinflammatory Drugs (NSAIDs) abuse, and co-morbidities such as underlying chronic liver disease, hypertension, and diabetes. Early endoscopy can be of great utility to reduce morbidity and mortality.

## Background

The incidence of UGIB is around 50–150 per 100,000 adults annually [1]. The older population was more commonly affected [2]. Hematemesis and/or melena are symptoms of UGIB, defined as bleeding from a source proximal to the Treitz ligament [3]. It might be anything from a significant hemorrhage to bleeding lasting for a few days with or without a change in hemodynamics. Bright red blood indicates recent hemorrhage. “Coffee ground” result from the influence of stomach acid on the blood. Melena is a black, tarry stool caused by upper GI hemorrhage. It relates to the impact of the GI tract and bacteria on the blood. Hematochezia is bright red blood in the stool, usually caused by a lower GI hemorrhage, but it can also occur due to a large, rapid UGIB. Colorectal bleeding is the most common cause of hematochezia, although it can also occur due to a large, rapid UGIB [4]. Increased mortality rate, recurrent bleeding, and the need for endoscopic hemostasis or surgery are associated with the risk factors such as age of more than 60 years, co-morbidities, active bleeding (e.g., witnessed hematemesis, coffee ground aspirate in nasogastric tube, fresh blood per rectum), hypotension, requirement for transfusion of more than or equal to six units of red blood cells [5]. There is no such study done in emergency department related to GI bleed in northern part of India, so we are doing this study to find out the clinical and epidemiological profile of adult patients with GI bleed presenting to emergency department.

## Materials and methods

### Study design and settings

This single-center, observational study of adult patients presenting with suspected UGIB to the emergency department of a tertiary care center was conducted with patient recruitment period from November 2020 to December 2021. All the patients aged 18 years and above presenting to the emergency department with suspected or confirmed UGIB were included in the study. The study was approved by the Institutional Ethical Committee, AIIMS Rishikesh. (Ref No.- AIIMS/ IEC/ 20/ 513).

### Sample size

We included all patients which comes to emergency department with suspected or confirmed UGIB during the specified study period and who fulfil inclusion criteria, so 141 patients were recruited by convenient sampling, after taking informed written consent from all the patients.

### Clinical evaluation

The study’s primary objective was to assess the socio-demographic and clinical characteristics of patients with suspected UGIB presenting to the emergency department. The secondary objective of the study was to assess the outcomes in patients with suspected UGIB presenting to the emergency department and to determine the association of clinical characteristics with outcomes among patients presenting to the emergency department with suspected UGIB. Detailed history, including demographic data such as age and sex, presenting complaints, history of presenting illness, history related to co-morbidities, addiction (such as recent alcohol intake within one week or chronic/regular alcohol consumption), and drug history, was obtained. Detailed physical and systemic examination was done in all the patients, and findings were noted. Laboratory tests obtained included total blood count, arterial blood gas, liver function tests, renal function tests, prothrombin time, international normalized ratio (INR), and activated partial thromboplastin time (aPTT). Shock index (Heart rate/Systolic Blood Pressure, HR/SBP) was calculated for each patient. Upper GI endoscopy was done in all the patients who are clinically suspected or confirmed to have UGIB. Upper GI endoscopy was performed on emergent basis for severe bleeding even at night and those patients with minor bleed endoscopy is mostly performed at daytime. As during our study period COVID-19 surge occurred so those patients whose gastric lavage was negative for blood were not considered for endoscopy by gastroenterology team.

Rockall and Blatchford’s score was calculated for each patient [6].

### Statistical analysis

The data was analyzed using SPSS software version 24. Categorical variables were presented in number and percentage (%), and continuous variables were presented as mean (SD) and median (Interquartile range, IQR) depending on the distribution of the data after assessing normality by the Shapiro-Wilk test. Categorical variables were analyzed with a chi-square test. The level of significance was set at p < 0.05. Continuous variables are analyzed using Spearman correlation coefficient and Pearson correlation coefficient. The receiver operating characteristic curves was plotted for each of the two scores and the area under the curves was analyzed.

## Result

Clinical evaluation was performed on 141 patients after obtaining written consent.

### Baseline characteristics of the study population

The mean age of the patients with suspected UGIB was 48 ± 14 years. 115(81.6%) patients were male. The most common co-morbidity was chronic liver disease (40;28.4%), followed by diabetes mellitus (19;13.5%), hypertension (20;14.2%), and 20(14.2%) patients who had a previous history of UGIB as shown in Table 1.

**Table 1:** Profile of socio-demographic parameters of patients with suspected UGIB.

Parameters	Mean ± SD
**Age**	48.62 ± 14.63
**Gender**	**No. (%)**
Male	115 (81.6)
Female	26 (18.4)
**Co-morbidities**	**No. (%)**
Hypertension	20 (14.2)
Type 2 Diabetes mellitus	19 (13.5)
Chronic liver disease	40 (28.4)
Alcohol use	68 (48.2)
Chronic kidney disease	10 (7.1)
Coronary artery disease	7 (5.0)
Previous UGIB	20 (14.2)
Antiplatelet use	8 (5.7)
NSAIDs use	9 (6.4)
Hepatitis C virus	12 (8.51)
Hepatitis B virus	5 (3.54)

NSAID Non Steriodal Anti Inflammatory Drugs, UGIB Upper Gastrointestinal Bleed

### Clinical profile of patients

The most frequent presenting complaint in this study was hematemesis (96; 68.1%) and melena (76;53.9%). 41(29%) patients complained of both hematemesis and melena. The mean shock index was 0.90 ± 0.29. 38(26.9%) patients had shock index > 1. The mean (SD) of the Rockall Score was 2.46 ± 1.75. The mean (SD) of the Glasgow Blatchford Score was 12.46 ± 3.15, as shown in Table 2.

**Table 2:** Clinical profile of patients with suspected UGIB presenting to the emergency department

Symptoms	No. (%)
Hematemesis	96 (68.1)
Malena	76 (53.9)
Hematemesis + Hematochezia	13 (9.2)
Recent alcohol binge	24 (17.0)
Syncope	1 (0.7)
Vomiting	28 (19.9)
Abdominal distension	48 (34.0)
Diarrhoea	4 (2.8)
Body pallor	15 (10.6)
Jaundice	29 (20.6)
Abdominal pain	50 (35.5)
Shortness of breath	10 (7.1)
Fever	12 (8.5)
Altered mental status	17 (12.1)
Anasarca	13 (9.2)
Decrease urine output	16 (11.3)
**Examination parameters**	**Mean ± SD**
Pulse rate (BPM)	98 ± 17
Shock index	0.90 ± 0.29
Rockall score	2 ± 1
Glasgow Blatchford score	12 ± 3

BPM Beats per minute

### Biochemical and hematological profile of patients

Majority of patients presented with anemia and hyperbilirubinemia in the study. The mean hemoglobin was 8.67 ± 2.7 g/dl, and the mean total bilirubin was 2.77 ± 3.7 mg/dl. 58 (41.1%) patients had coagulopathy. The mean prothrombin time was 20.95 ± 16.03 s, and the mean INR was 1.65 ± 0.73 s. The mean lactate level was 2.69 ± 2.60 mmol/l, and the anion gap was 13.32 ± 5.82. Biochemical and hematological profile of patients with suspected UGIB are shown in Table 3.

**Table 3:** Biochemical and hematological profile of patients with suspected UGIB.

Parameters	Mean ± SD
Hemoglobin (g/dL)	8.6 ± 2.6
Hematocrit (%)	26.4 ± 7.3
Platelet Count (x10³/μL)	129.5 ± 74.2
Total Leucocyte Counts (x10³/μL)	9.5 ± 5.7
Urea (mg/dL)	63.7 ± 58.8
Creatinine (mg/dL)	1.6 ± 2.2
Total Bilirubin (mg/dL)	2.7 ± 3.7
Prothrombin Time (s)	20.9 ± 16.0
International Normalized Ratio	1.6 ± 0.7
Aspartate aminotransferase (U/L)	126.8 ± 351.8
Alanine aminotransaminase(U/L)	57.5 ± 74.3
Alkaline phosphatase (U/L)	214.3 ± 134.1
pH	7.39 ± 0.08
HCO_3_ (mmol/L)	18.9 ± 4.5
Lactate (mmol/L)	2.6 ± 2.6
Anion Gap	13.3 ± 5.8

### Upper GI endoscopy/colonoscopy findings

Upper GI endoscopy was done only in 104 out of 141 patients, as during our study period COVID-19 surge occurred so those patients whose gastric lavage was negative for blood were not considered for endoscopy by gastroenterology team. Eight patients (three hemorrhoids, two ulcerative colitis, and three no obvious source of bleed) underwent a colonoscopy simultaneously. This was done because the upper GI endoscopy did not show any bleeding source in those patients. Esophageal varices (55;52.9%) were the most common finding, followed by gastric ulcer (12;11.5%), duodenal ulcer (6;5.8%), esophageal ulcer (6;5.8%), esophagitis (5;4.8%), and gastric carcinoma (2;1.9%). Thus, portal hypertension was seen in more than 50% of cases of UGIB in our study, as shown in Table 4.

**Table 4:** Upper GI endoscopy/colonoscopy findings

UGI endoscopy / Colonoscopy findings	No. (%)
Esophageal varices	55 (52.9)
Gastric ulcer	12 (11.5)
Duodenal ulcer	6 (5.8)
Esophageal ulcer	6 (5.8)
Esophagitis	5 (4.8)
Post EVL ( endoscopic variceal ligation) ulcer	3 (2.9)
Hemorrhoids	3 (2.9)
Ulcerative colitis	2 (1.9)
Carcinoma stomach	2 (1.9)
Ulcer at the gastroesophageal junction	2 (1.9%)
Candidiasis	1 (1.0%)
Gastropathy	1 (1.0%)
Alcohol-induced gastritis	1 (1.0%)
Mallory Weiss tear	1 (1.0%)
Esophageal diverticulum	1 (1.0%)
No active source of the bleed	3 (2.9%)
Normal study	6 (5.8%)

EVL Endoscopic Variceal Ligation

### Treatment profile and outcome of patients with suspected UGIB

In our study majority of patients (108;77.7%) received intravenous fluids (crystalloids), 37 (26.6%) patients received packed red blood cells, 13(9.4%) patients received fresh frozen plasma, whereas only three (2.2%) patients received random donor platelets. Upper GI endoscopy was done in (104,73.75%) patients. Endoscopic variceal ligation was done in 35(33.7%) patients to control active bleeding. 8 (5.8%) patients were intubated in emergency department because of poor Glasgow Coma Score (GCS) (< 8).

Out of all 141 patients presenting with suspected UGIB, 93(66.0%) patients got admitted. 24(25.8%) patients were admitted to Intensive Care Unit (ICU) out of total admitted. Among 22(15.6%) patients who succumbed to death, four (18%) expired in the emergency department, five (22.7%) deaths occurred within 24 h, and 16(72.72%) within seven days after admission. Among patients admitted to ICU, 18(75%) succumbed to death, one(0.07%) patient left against medical advice, as shown in Table 5.

**Table 5:** Treatment profile and outcome of patients with suspected UGIB

Treatment		No. (%)
Endoscopy with or, without endotherapy		104 (73.75%)
Endotracheal intubation		8 (5.8%)
Intravenous fluids		108(77.7%)
Blood transfusion	PRBC	37 (26.6%)
	FFP	13 (9.4%)
	RDP / platelets	3 (2.2%)
Endoscopic variceal ligation		35 (33.7%)
**Outcomes**		**No. (%)**
Admission		93 (66.0%)
ICU admission out of total admitted		24 (25.8%)
Discharge		43 (30.5%)
Mortality (out of those admitted in ICU)		18 (75%)
Mortality (in hospital)		22 (15.6%)
Mortality in emergency department		4 (18%)
Mortality in 24 h		5 (22.7%)
Mortality in 7 days		16 (72.72%)
Left against medical advice		1 (0.7%)

UGIE Upper Gastrointestinal Endoscopy, PRBC Packed Red Blood Cells, FFP Fresh Frozen Plasma, RDP Random Donor Platelet

### Cause of mortality in patients with UGIB in emergency department

Septic shock (13;61.9%) was the most common cause of mortality. Septic shock is diagnosed as per ‘Sepsis 3’ definition i.e. ‘any patient who fulfill the criteria for sepsis who, despite adequate fluid resuscitation, require vasopressors to maintain a mean arterial pressure (MAP) more than or equal to 65mmHg and have a lactate more than 2 mmol/L’. It is followed by metabolic acidosis (3;14.3%), hypovolemic shock (2;9.5%), and acute respiratory distress syndrome (2;9.5%), as shown in Table 6.

**Table 6:** Cause of mortality in patients with UGIB in emergency department

Causes	No. (%)
Septic shock	13 (61.9)
Hypovolemic shock	2 (9.5)
Metabolic acidosis	3 (14.3)
ARDS (acute respiratory distress syndrome)	2 (9.5)
Ventricular fibrillation	1 (4.8)
AKI with Septic shock	1 (4.8)

AKI Acute Kidney Injury

### Correlation between the Rockall score and Glasgow Blatchford score with outcome parameters

The mean duration of emergency department stay was 12.45 ± 11.78 h.

The mean hospital stay of patients with UGIB was 6.27 ± 5.03 days.

Table 7 shows the correlation between the Rockall score and Blatchford score and outcome parameters such as length of hospital stay (days), length of emergency department stay (hours), and blood transfusion. There was no significant association between Rockall and Blatchford score and hospital stay. Rockall and Blatchford scores were significantly associated with the need for blood transfusion and products.

**Table 7:** Correlation between the Rockall score and Glasgow Blatchford score with outcome parameters

Parameter	Rockall score	Glasgow Blatchford score
	Spearman Correlation Coefficient (P Value)	Spearman Correlation Coefficient (P Value)
Length of hospital stay (days)	-0.1 (0.622)	0.1 (0.403)
Length of emergency department stay (hours)	-0.1 (0.389)	-0.1 (0.539)
**Blood transfusion**	**Point-Biserial Correlation (P Value)**	**Point-Biserial Correlation (P Value)**
PRBC	0.25 (0.012)	0.47 (< 0.001)
FFP	0.16 (0.065)	0.27 (< 0.001)
RDP	0.22 (0.015)	0.14 (0.060)

PRBC Packed Red Cells, FFP Fresh Frozen Plasma, RDP Random Donor Platelet

### Association between clinical parameters with mortality

There was a significant association between mortality and clinical variables such as high respiratory rate, low SpO2, high pulse rate, low systolic blood pressure, low diastolic blood pressure, low GCS, and high shock index. Also, hematological and biochemical parameters, such as low hemoglobin, low hematocrit, low platelet count, high total leucocyte count, high lactate level, elevated blood urea, and serum creatinine level, were significantly associated with mortality, as shown in Table 8.

**Table 8:** Association between clinical parameters with mortality

Parameters	Death (n = 22)	Survival (n = 119)	p value
Respiratory rate	21.20 ± 2.71	20.18 ± 2.64	0.042
SpO2(%)	91.64 ± 15.19	97.36 ± 2.54	0.027
Pulse rate	109.86 ± 11.33	96.27 ± 17.19	< 0.001
Systolic blood pressure	95.59 ± 33.79	115.08 ± 21.63	< 0.001
Diastolic blood pressure	66.07 ± 16.51	74.21 ± 12.39	0.009
GCS	12.82 ± 3.67	14.50 ± 1.66	0.010
Shock index	1.18 ± 0.27	0.86 ± 0.27	< 0.001
Endotracheal Intubation	4 (18.2%)	4 (3.4%)	0.022
Hemoglobin(g/dl)	7.21 ± 2.35	8.94 ± 2.67	0.004
Hematocrit (%)	22.45 ± 6.56	27.15 ± 7.29	0.004
Platelet Count (x10³/μL)	93.46 ± 71.05	136.21 ± 73.13	0.001
Total leucocyte count(x10³/μL)	13.81 ± 6.43	8.75 ± 5.31	< 0.001
Bicarbonate(mmol/l)	16.47 ± 5.04	19.44 ± 4.37	0.015
Lactate(mmol/l)	5.21 ± 4.25	2.22 ± 1.84	< 0.001
Anion gap	15.46 ± 4.78	12.93 ± 5.93	0.008
Urea(mg/dl)	77.39 ± 46.93	61.27 ± 60.58	0.021
Creatinine(mg/dl)	2.08 ± 1.37	1.60 ± 2.34	0.006
Prothrombin time(s)	27.86 ± 12.43	19.67 ± 16.34	< 0.001
INR	2.37 ± 1.00	1.52 ± 0.59	< 0.001
Aspartate aminotransferase(U/L)	244.53 ± 584.57	105.13 ± 288.06	0.016
Rockall score	3.45 ± 1.63	2.28 ± 1.72	0.005
Glasgow Blatchford Score	14.82 ± 2.17	12.03 ± 3.12	< 0.001

GCS Glasgow Coma Scale, INR International Normalised Ratio

**Fig. 1:**
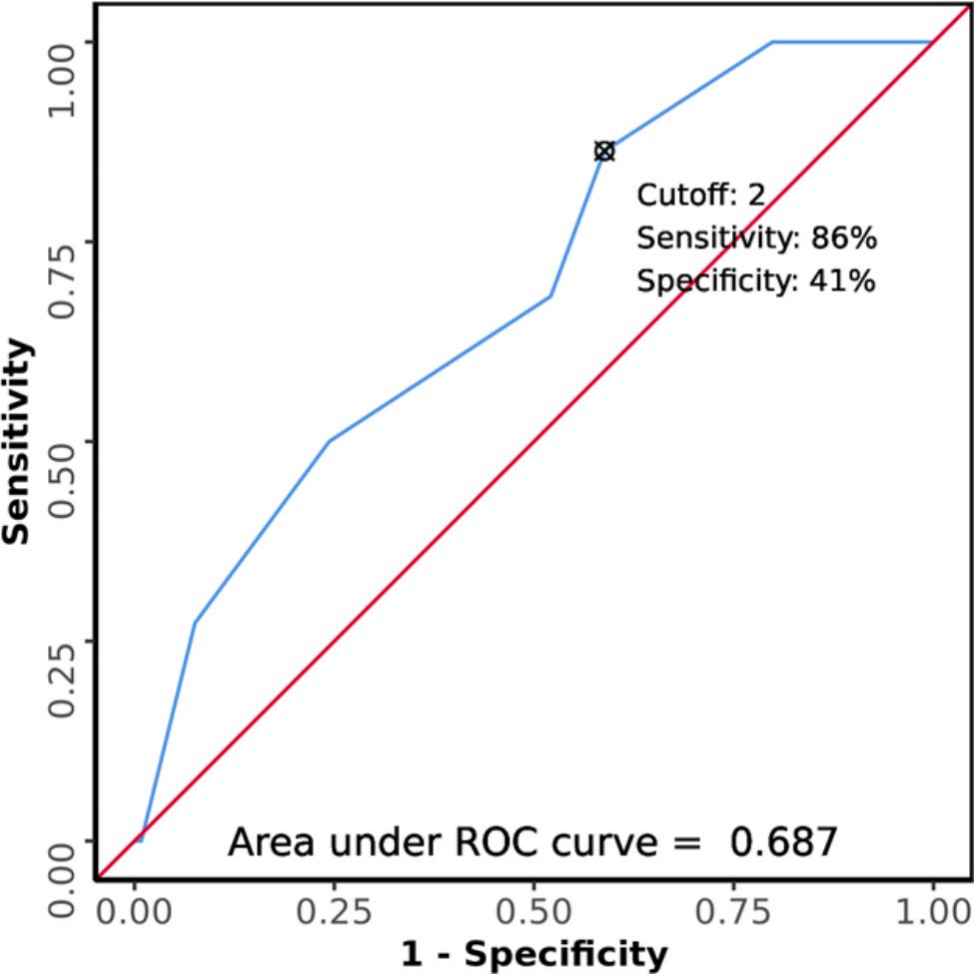
ROC Curve Analysis Showing Diagnostic Performance of Rockall Score in Predicting Outcomes (n = 141)

Figure 1 shows the area under the Receiver Operating Characteristic curve (AUROC) for Rockall Score predicting mortality was 0.687 (95% CI: 0.574–0.801). It was statistically significant (p = 0.005). At a cutoff of Rockall Score ≥ 2, it predicted death, with a sensitivity of 86% and a specificity of 41%.

**Fig. 2:**
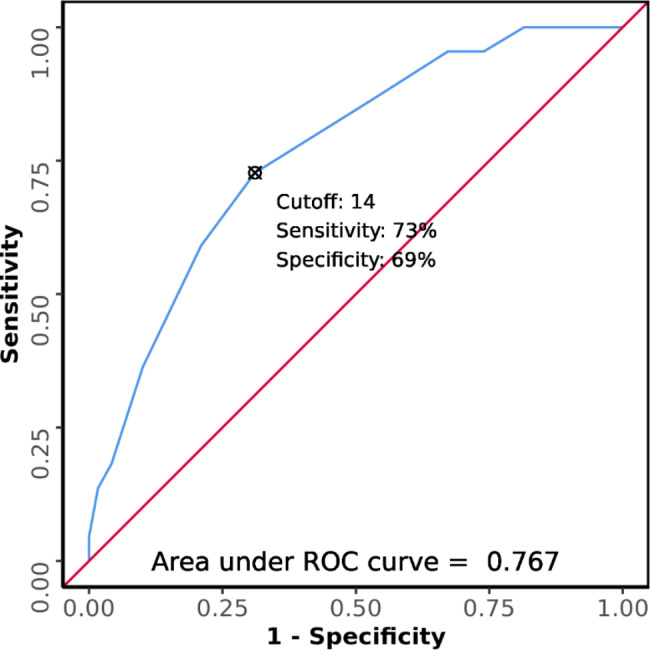
ROC Curve Analysis Showing Diagnostic Performance of Glasgow Blatchford Score in Predicting Outcomes (n = 141)

Figure 2 shows the area under the ROC curve (AUROC) for Glasgow Blatchford Score predicting outcomes was 0.767 (95% CI: 0.665–0.868). It was statistically significantly associated with mortality (p = < 0.001). At a cutoff of Glasgow Blatchford Score ≥ 14, it predicted mortality with a sensitivity of 73% and a specificity of 69%.

## Discussion

An UGIB is a potentially life-threatening medical emergency. These patients either come with frank hematemesis or melena [7]. They also visit the primary family physician. The etiology of UGIB can be subdivided into variceal and non-variceal bleeding [6]. The most common etiology of UGIB is gastric ulcer, followed by variceal bleeding in patients with alcoholic liver disease. The approximate incidence of UGIB is 100–200 cases per 1 lakh population. Around 65–80% of all bleeding incidents occur in the upper intestine [8].

### Demographic parameters

In our study, males dominated with mean age of presentation (48.62 ± 14.63 years). A cross-sectional study carried out by Surendran M et al. at a tertiary care hospital in southern India found that males (107;77.5%) outnumbered females (31;22.5%) with an average age of 53.5 ± 13.17 years [9]. Similar results were reported by Shenoy V et al. [10]. It is discernable from the above studies that UGIB is more prevalent in men than in women. In India, it has been observed that alcohol use disorder is more prevalent among males, which is a major cause of variceal bleeding in these patients [11].

### Co-morbidities

Chronic liver disease (40;28.4%) was the most prevalent co-morbidity in our analysis, followed by chronic renal disease (10;7.1%), chronic hepatitis C (12;8.51%), hepatitis B (5;3.54%), hypertension (20;14.2%), and type 2 diabetes (19;13.5%). According to the previous study conducted by Mahajan et al., a significant association was observed between co-morbidities such as diabetes mellitus, coronary artery disease, and mortality [12]. Another study by Bhattarai et al. observed that 45.5% of patients with UGIB had chronic liver disease, 3.7% had chronic hepatitis B, and 1.5% had hepatitis C, which was comparable with our study [13]. It has been observed by a previous study that chronic liver disease was more commonly seen in patients with variceal bleeding, whereas co-morbidities like cardiac disease, cerebrovascular accidents, and malignancies were more prevalent in patients with non-variceal bleeding [14]. Thus, co-morbidities screening is important in patients with UGIB as they add to the risk of mortality in these patients.

### Clinical features

Hematemesis was the most prevalent presenting symptom in UGIB patients in our research, followed by melena, discomfort abdomen, abdominal distension, and both hematemesis and melena. Minakari M et al. found that hematemesis (63.5%) was the most common presentation [15]. Similar results were reported by Rajan ss et al. and Shah H et al. [6, 16]. This hospital is a tertiary care and referral center. It caters to the majority population of Uttarakhand and neighbor states. Most of the patients have a rural background. Often, the patients do not notice the initial symptoms and signs of melena and visit the hospital when massive hematemesis occurs.

### Laboratory parameters

Studies performed by Bhattarai et al. reported anemia in the majority of patients with UGIB similar to our study [13]. Identical results were reported by Sharma V et al. [17]. Bressler B et al. observed that the formation of esophageal varices is associated with thrombocytopenia (< 200,000/mm^3^) and hyperbilirubinemia (> 20 μmol/l). This study also concluded that if the patient has no history of UGIB and the laboratory report shows these type of values, then they should be screened for esophageal varices [18]. Anemia is common in patients with UGIB due to ongoing blood loss. Patients with chronic liver disease may have underlying megaloblastic anemia and pancytopenia due to hypersplenism. Urea and creatinine are raised in these patients as a result of blood being metabolized into protein, and this protein is transported to the liver, where it is converted to blood urea nitrogen. Blood urea nitrogen increases due to the decrease in hydration and hypovolemia. Acute UGIB can cause a rise in blood urea nitrogen and creatinine ratios, as well as a reduction in renal perfusion.

### Upper GI endoscopy findings

There have been earlier research with similar results to ours like Surendran M et al. found that esophageal varices (51.4%) were the most common finding, followed by gastritis (15.2%) [9]. Paudal MS et al. observed that duodenal ulcer, present in 29% cases was the most common finding, followed by varices in 23% cases [19]. In contrast to our study, Kashyap R et al. observed that peptic ulcer was present in 61% of patients [20]. This discrepancy can be attributed to more prevalence of alcoholic liver disease in our state.

### Interventions

There are many treatment modalities for acute UGIB. Injections of epinephrine (1:10 000 dilution), injections of sclerosants such as 100% ethanol, thermal contact devices such as bipolar electrocoagulation probes or heater probes, and clips are recommended for bleeding ulcers. Shenoy V et al. observed that PRBC transfusion was done in 46.7% cases whereas 37(26.6%) cases received PRBC transfusion in our study [10]. JP Hreinsson et al. found that 60% of patients presented with UGIB required blood transfusion [21]. In O’Donnell’s study, one hundred (1.5%) patients developed gastrointestinal haemorrhage during hospitalisation following an acute ischemic stroke, with 36 (0.5%) requiring blood transfusion [22].

### Outcomes

In our study 141 patients presented with suspected UGIB, and 93 (66.0%) patients were hospitalized. Similarly, J P Hreinsson et al. found that out of 156 patients with UGIB 71% were hospitalized [21].

Thus, it has been observed that the majority of patients with acute UGIB require hospitalization for hemodynamic stabilization and immediate intervention.

A study conducted by Rajan et al. observed that 34.8% of patients with UGIB succumbed to death within 24 h of presentation, and 18.7% succumbed in seven days [6]. Moledina S et al. found that around 24.6% of patients succumbed within 24 h of admission, and 49.1% died within 72 h after admission [23]. As compared to previous studies more number of patients died in our study. This highlights the importance of early intervention, which can significantly improve the outcome of patients with UGIB.

In our study, the mean emergency department stay of patients with UGIB was 12.45 ± 11.78 h, and the mean hospital stay was 6.27 ± 5.03 days. According to the study conducted by Mungan Z et al., the average length of hospital stay was 5.63 days with a standard deviation of 4.91 days [24]. Thus, it has been observed that these patients with UGIB require immediate stabilization and intervention. These patients after stabilization were placed on intravenous octreotide infusion or terlipressin injection for 48 to 72 h as per guidelines.

In our study, the area under the ROC curve (AUROC) for Rockall Score with mortality prediction was 0.687 (95% CI: 0.574–0.801). Sharma V et al. observed that the clinical Rockall score (AUROC = 0.677, 95% CI: 0.583–0.770, p = 0.001) was useful in predicting mortality [17]. This was comparable to our study findings.

UGIB is one of the most common emergency condition. These patients present to family medicine physicians as well as emergency physicians. UGIB can have varied etiology such as gastric ulcer, chronic liver disease, and coagulopathies. Immediate priorities should be given to these patients to stabilize these patients primarily, early upper GI endoscopy should be done, and appropriate intervention should be planned. The primary care physician and the emergency medicine physician should actively manage these patients.

One potential drawback of our study is that it was conducted at a single centre and was time bound. The sample size was determined by convenient sampling, which was another limitation. Because the study was completed during the COVID 19 surge, a lesser number of patients were enrolled.

## Conclusion

Portal hypertension is one of the most important causes of UGIB. Early intervention should be planned in these patients to prevent mortality and morbidity.

In our study, hematemesis was the most prevalent symptom of suspected UGIB followed by melena. Around a third of patients required blood transfusion. Most frequent comorbidities in patients suspected of UGIB were alcohol intake, NSAIDs abuse, and co-morbidities such as underlying chronic liver disease, hypertension, and diabetes. Early endoscopy can be a solution to reduce morbidity and mortality. Thus, awareness about the etiology, associated risk factors, and predictors of mortality in patients with UGIB can help the emergency duty physicians to control the acute UGI bleeding and counsel the patients in taking preventive measures at appropriate time, without any inappropriate delay, which can be detrimental to the patient.

## Data Availability

The datasets used and/or analyzed during the current study are available from the corresponding author on reasonable request.

## Abbreviations

aPTT: Activated Partial Thromboplastin Time
AKI: Acute Kidney Injury
ARDS: Acute Respiratory Distress Syndrome
AUROC: Area Under the Receiver Operating Characteristic Curve
ED: Emergency Department
EVL: Endoscopic Variceal Ligation
FFP: Fresh Frozen Plasma
GCS: Glasgow Coma Score
GI: Gastrointestinal
HR: Heart rate
ICU: Intensive Care Unit
INR: International Normalised Ratio
IQR: Interquartile Range
NSAID: Non Steriodal Anti Inflammatory Drugs
PRBC: Packed Red Blood Cells
RDP: Random Donor Platelet
SBP: Systolic Blood Pressure
SD: Standard Deviation
UGIB: Upper Gastrointestinal Bleed
UGIE: Upper Gastrointestinal Endoscopy
